# Exploring Calcium Channels as Potential Therapeutic Targets in Blast Traumatic Brain Injury

**DOI:** 10.3390/ph18020223

**Published:** 2025-02-06

**Authors:** Noemi Wachtler, Rory O’Brien, Barbara E. Ehrlich, Declan McGuone

**Affiliations:** 1 School of Medicine and Health, Technical University of Munich, 81675 Munich, Germany; noemi.wachtler@yale.edu; 2 Department of Pharmacology, Yale School of Medicine, New Haven, CT 06510, USA; 3 Department of Neurology, Yale School of Medicine, New Haven, CT 06510, USA; 4 Department of Pathology, Yale School of Medicine, New Haven, CT 06510, USA

**Keywords:** traumatic brain injury, calcium signaling, excitotoxicity, mitochondrial dysfunction, neurodegeneration, calcium channel inhibitors, blast injury, mechanoporation

## Abstract

**Background/Objectives**: Repeat low-level blast exposure has emerged as a significant concern for military populations exposed to explosive events. Blast-Related Traumatic Brain Injury (bTBI) is a unique form of brain trauma with poorly understood molecular mechanisms. Loss of calcium homeostasis has emerged as a mediator of early neuronal dysfunction after blast injury. This review aims to examine the role of calcium signaling in bTBI, focusing on the dual function of calcium channels as mediators and modulators of injury, and to explore therapeutic strategies targeting calcium homeostasis. **Methods**: We conducted a review of peer-reviewed articles published between 2000 and 2024, using the databases PubMed, Scopus, and EBSCO. Search terms included “blast traumatic brain injury”, “calcium channels”, and “calcium”. Studies investigating intracellular calcium dynamics after bTBI were included. Exclusion criteria included studies lacking evaluation of calcium signaling, biomarker studies, and studies on extracellular calcium. **Results**: We identified 13 relevant studies, primarily using preclinical models. Dysregulated calcium signaling was consistently linked to cellular dysfunction, including plasma membrane abnormalities, cytoskeletal destabilization, mitochondrial dysfunction, and proteolytic enzyme activation. Studies highlighted spatially compartmentalized vulnerabilities across neurons and astrocytes, suggesting that targeting specific cellular regions, such as the neuronal soma or axons, could enhance the therapeutic outcome. Therapeutic strategies included pharmacological inhibitors, plasma membrane stabilizers, and modulators of secondary injury. **Conclusions**: Calcium signaling is implicated in the pathophysiology of bTBI. Standardized experimental approaches would reduce variability in findings and improve the understanding of the relationship between calcium channel dynamics and bTBI and help guide the development of neuroprotective interventions that mitigate injury and promote recovery.

## 1. Introduction

Traumatic brain injury (TBI) is a leading cause of long-term disability and mortality among young people in high-income countries, with approximately 60 million cases reported worldwide each year [1,2]. In military settings, TBI has been termed the “signature wound” of modern warfare, with an estimated 414,000 combat-related head injuries reported by the United States Defense and Veterans Brain Injury Center between 2000 and 2019 [3,4]. Most of these injuries are classified as mild TBI, clinically akin to concussion, and are predominantly associated with blast exposures [5,6]. Unlike penetrating or blunt force head trauma, blast-related TBI (bTBI) results from rapid changes in atmospheric pressure generated by explosive energy release, creating a blast overpressure (BOP) wave followed by underpressure [7,8]. These forces initiate complex interactions with the brain, and although the long-term neuropathological sequelae of bTBI are incompletely understood, primary blast waves are a central focus of bTBI research [7,9].

The recognition that bTBI could impair neuropsychological function without evident brain damage dates back to World War I, when a constellation of symptoms such as tremor, amnesia, poor concentration, and hypersensitivity to noise were collectively termed “shell shock”, which resembles the modern understanding of post-traumatic stress disorder (PTSD) [10,11]. In modern conflicts, bTBI prevalence has surged, driven by exposure to high-energy improvised explosive devices and advanced munitions [12,13,14]. Recently, attention has shifted to repeat low-level blast (LLB) exposures, common in military training scenarios routinely encountered by breaching instructors and artillery operators [15]. Unlike mild TBI (mTBI), LLB exposure does not cause concussion, although it has been linked to cognitive, behavioral, and neuropathological effects, including white matter and blood–brain dysfunction, with unclear impacts on motor, neurosensory, and visual function [16,17]. Auditory impairments like tinnitus and hearing loss are common, and behavioral symptoms including problematic anger, impaired impulse control, anxiety, sleep disturbance, and depression are described [18,19,20,21,22]. Unlike conventional TBI, the symptoms of LLB exposure emerge gradually, complicating diagnosis and treatment. Chronic underreporting due to misattribution of symptoms and career concerns severely hampers accurate estimates of disease prevalence and impact [23].

This review focuses on cellular and molecular changes following bTBI, with a specific emphasis on calcium channels and dysregulated calcium signaling after injury. Calcium, a pivotal mediator of biological signals, is central to cellular metabolism, gene expression, neurotransmission, and synaptic plasticity [24,25,26,27]. Loss of calcium homeostasis, a hallmark of TBI, activates multiple secondary injury mechanisms linked to brain edema, microvascular injury, blood–brain barrier disruption, ischemia, excitotoxicity, mitochondrial dysfunction, axonal injury, neurodegeneration, and inflammation [28,29,30,31,32]. There are diverse calcium channels that, upon dysregulation, can influence the cellular calcium homeostasis, some of which are voltage-gated calcium channels (VGCS), mechanosensitive channels (MSC), ligand-gated channels, and intracellular channels like inositol trisphosphate receptors (IP3Rs) and ryanodine receptors (RyRs). Understanding how these mechanisms become deregulated after bTBI is critical for developing neuroprotective strategies that mitigate the long-term risks of repeat LLB exposure.

## 2. Literature Search and Methodology

We performed a systematic search of electronic databases including PubMed, Scopus, Google Scholar, ProQuest, and EBSCO for articles published between 2000 and 2024. The search strategy combined the terms “blast traumatic brain injury”, “calcium channels”, and “calcium” as keywords or MeSH terms to identify peer-reviewed original research articles in English. Relevant articles were reviewed and duplicates removed. Due to the limited number of relevant studies, a meta-analysis would have been impractical and potentially misleading. No randomized control trials or cohort studies evaluating calcium channel therapies after blast exposure in humans were identified. Consequently, this review is limited to preclinical and animal model studies explicitly investigating intracellular calcium in bTBI. We excluded review articles; studies describing calcium changes in serum, cerebrospinal fluid, or extracellular calcium; biomarker studies focusing on proteins with calcium binding properties (e.g., S100B protein); morphological studies that used ionized calcium-binding adapter molecule 1 (Iba1) antibodies to label microglia; and bTBI studies that did not mention calcium.

After duplicates were removed and papers were screened, thirteen relevant papers were identified, three of which originated from the same research group (Table 1).

**Figure 1:**
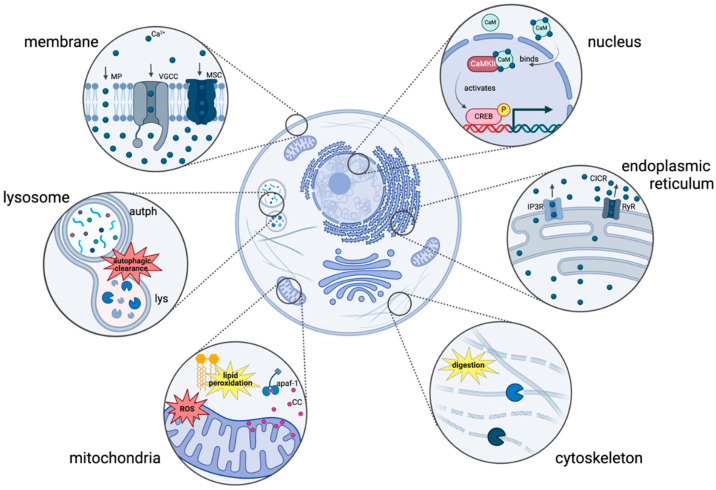
Mechanisms of cellular and molecular dysregulation in blast-related traumatic brain injury (bTBI). Vulnerable areas are highlighted as injury hotspots. Membrane: disruption of membrane permeability occurs through mechanoporation (MP), calcium influx through membrane pores, voltage-gated calcium channels (VGCCs), and mechanosensitive channels (MSC). Lysosome: impaired fusion between autophagososme (autph) and lysosome (lys) disrupts autophagic clearance. Mitochondria: production of reactive oxygen species (ROS) facilitates lipid peroxidation of the lipid bilayer and release of cytochrome C (CC; binds to apaf-1 to induce formation of the apoptosome and apoptosis) into the cytoplasm. Nucleus: calmodulin (CaM) buffers calcium upon rise in intracellular concentration; CaM-bound calmodulin kinase II (CaMKII) activates transcription factors. Endoplasmic reticulum: calcium-induced calcium release (CICR) activates ryanodine receptors (RyRs), and inositol 1,4,5 trisphosphate (IP3) production activates IP3 receptors (IP3Rs). Cytoskeleton: calcium-induced activation of calpain and other calcium-dependent enzymes leads to cytoskeletal degradation. Image created in https://BioRender.com.

To understand the impact of blast exposure on calcium channels and calcium homeostasis, the results are organized by cellular compartments: plasma membrane, cytoskeleton, endoplasmic reticulum (ER), mitochondria, nucleus, and lysosomes (Figure 1).

## 3. Cellular and Molecular Pathophysiology of Blast TBI

### 3.1. Plasma Membrane and Calcium Dysregulation After Blast Exposure

The biomechanical forces associated with LLB exposure result in plasma membrane deformation and increased membrane permeability [45]. The plasma membrane is a central mediator of early responses to trauma, but the extent to which these changes are reversible after blast exposure remains to be determined. Increased intracellular calcium resulting from membrane deformation, mechanoporation, and altered ion flows is well described in non-blast TBI, including focal and diffuse injury, with recovery of calcium homeostasis often prolonged [46,47,48,49,50]. Similar disruptions in membrane permeability are observed using in vitro models of neuronal stretch [51]. Primary mechanoporation occurs within milliseconds of injury, forming transient pores that disrupt ionic gradients and facilitate unregulated calcium influx [52]. The initial pore-forming event may be reversed by membrane-resealing mechanisms, or it can be followed by the formation of secondary pores which develop hours later through calpain activation [53,54]. Mechanoporation disrupts normal action potential initiation and propagation in a spatial and dose-dependent manner, and at the same time, the membrane pores facilitate direct excitotoxicity independent of transporter-mediated calcium entry [55,56].

Additionally, mechanical strain can disrupt plasma membrane bound protein channels, such as voltage-gated sodium channels (VGSCs), which are sensitive to mechanical stress [57]. VGSC activation leads to cell membrane depolarization, triggering voltage-gated calcium channels (VGCCs) and initiating a pathological feedback loop of calcium signaling. The release of excitatory amino acids such as glutamate amplifies calcium dysregulation through activation of calcium-permeable α-amino-3-hydroxy-5-methyl-4-isoxazolepropionic acid (AMPA) receptors and N-methyl-D-aspartate (NMDA) receptors. NMDA receptor activation after trauma results in excitotoxicity, ionic imbalances, neuronal depolarization, and cortical spreading depression [56,58]. Cortical spreading depressions complicate up to 60% of moderate to severe non-blast TBIs and can precipitate a metabolic crisis in the brain [59,60]. Interestingly, NMDA receptors may also be mechanically activated in a glutamate-independent manner, though the relevance of this mechanism to bTBI is unclear [61,62].

Multiple in vitro studies have demonstrated the importance of mechanical stress for initiating dysfunctional calcium signaling after blast exposure. One in vitro model of single and repetitive primary blast injury (10–14 psi) using neurons and mixed cell cultures found a dose-dependent increase in membrane permeability to sodium without significant changes in intracellular calcium [45]. In this model, the cells were fixed to a rigid substrate, which may have acted as a confounder by preventing sufficient shear strain to drive the calcium influx. Neuronal and glial cells exposed to repetitive blast conditions exhibit increased fluorescence with the membrane-impermeable dye calcein, indicating mechanoporation. Excessive glutamate release after repeat-blast injury was not accompanied by significant elevations in intracellular calcium, suggesting that glutamate-dependent excitotoxicity is unlikely to be a major mechanism of injury in isolated cells exposed to repeated blasts. Although changes in membrane permeability following LLB exposure are generally transient, a rapid influx of sodium can induce osmotic stress, leading to cellular edema, cytoskeletal breakdown, and axonal spheroid formation [38,63]. A different model using a compressed air-driven shock tube to deliver higher pressure blasts (21 psi) to NG108-15 hybrid cells or SH-SY5Y human neuroblastoma cells found decreased intracellular ATP, increased lactate dehydrogenase (LDH) release into culture medium, and no significant changes in intracellular calcium [64]. In primary human neuronal cultures, calcium changes correlate better with the magnitude of shear strain than the intensity of blast overpressure, again underscoring the importance of shear strain as a necessary condition for initiating changes in intracellular calcium after blast injury [41].

In contrast to repetitive LLB, primary neuronal and astrocytic cultures subjected to higher magnitude blast strain or direct mechanical injury exhibit mechanoporation combined with disruptions in calcium homeostasis [34,37,65]. Poloxamer 188 (P188), an FDA-approved copolymer known for sealing mechanoporation in biomembranes, has therapeutic efficacy in sealing membrane damage in both in vitro and in vivo models of non-blast TBI [66,67,68]. In blast injury, P188 can restore physiological function after stretch or blast injury [34,37,65]. For example, primary chick forebrain neurons subjected to fluid shear stress show a gradual increase in intra-axonal calcium lasting up to 30 min post-injury, which can be blocked by administration of P188 within 5 min of injury [65]. Similarly, mouse astrocytes exposed to microcavitation (collapse of microbubbles generated by pressure changes) exhibit large, transient increases in intracellular calcium, followed by a steady decline without the spontaneous spiking activity, and this phenotype can be partially reversed by early post-injury treatment with P188 [34,37]. These findings suggest that neurons and astrocytes experience mechanoporation following simulated higher-magnitude blast strain in vitro, leading to disrupted calcium homeostasis, and that this can be rescued by timely treatment with P188.

Translational animal models of bTBI have relied on shock tubes, blast tubes, and open-field explosives; however, significant variation in experimental design, including shock tube specifications, choice of anesthetic, species tested, orientation of the animal relative to the blast wave, and multiple variations in blast intensity and frequency, have led to striking inconsistencies in behavioral outcomes among laboratories [69]. One study that used a cranium-only blast injury model (peak BOP of 1399 ± 252 KPa) to assess the effect of LLB on unanesthetized mice reported strikingly heterogeneous changes in intracellular calcium dynamics in hippocampal neurons [36]. In this experiment, most injured neurons had significant and sustained decreases in basal intracellular calcium, accompanied by a reduced frequency of calcium transients that returned to baseline within one hour. A smaller subset of neurons showed sustained elevations in basal calcium, and a few cells displayed no significant changes. These changes were accentuated upon exposure to a second blast. Interestingly, these blast-induced changes in basal calcium did not correlate well with changes in rapid calcium transients. Despite the changes in cellular calcium, none of the blast-exposed animals lost consciousness or demonstrated abnormal behavior within the first week post-injury, supporting appropriate classification as an LLB exposure model. Although LLB exposure appeared to modulate neuronal function at a network level, the study did not classify the excitatory or inhibitory characteristics of the neuronal populations affected. Thus, it remains unclear which hippocampal neuron subpopulations show increased basal calcium post-blast and how this relates to long-term cognitive dysfunction and neurodegenerative risk, given the importance of the hippocampus in memory formation and Alzheimer’s disease.

Beyond mechanoporation, mechanosensitive calcium channels like Piezo 2 also appear to contribute to calcium dysregulation after LLB exposure. In microfluidic flow chamber models, mechanosensitive channel inhibitors such as gadolinium (Gd^3+^) effectively reduce calcium influx in astrocytes exposed to simulated LLB injuries [40]. In vivo, rats subjected to moderate pressure free-field blasts exhibited increased mRNA expression of Piezo 2, whereas higher-pressure blasts did not elicit the same response [44]. Similarly, astrocytes exposed to pneumatic device-generated blast shockwaves showed elevated intracellular calcium levels only under conditions that involved shear forces, even with minimal pressure transients present, again underscoring the role of mechanical shear in activating calcium channels [41,43]. The prevailing hypothesis suggests that mechanosensitive channels, lipid pores, purinergic ATP channels, or a combination of these mechanisms contribute to calcium dysregulation in vulnerable cell populations, and that these changes are amplified through cell-to-cell signaling [41]. Astrocytes, which form an interconnected network that accounts for up to 40% of the total human brain mass, play a critical role in mediating this process, and astrocytic purinergic signaling has been shown to propagate calcium waves in dissociated human CNS cultures after blast injury [42,70,71]. Blocking P2 receptors with pyridoxalphosphate-6-azophenyl-2′,4′-disulfonic acid (PPADS) blunts this response [42]. In blast-injured astrocytes, post-injury treatment with ω-conotoxin, an N-type VGCC inhibitor restored calcium signaling, whereas verapamil or nickel, non-specific VGCC inhibitors, had no effect, suggesting that blocking N-type calcium channels may have neuroprotective effects after LLB [34]. Interestingly, treatment with P188, a membrane repair agent, also rescued calcium signaling through a mechanism unrelated to N-type channels. Combining ω-conotoxin and P188 did not result in synergistic effects, indicating that these drugs act through separate pathways.

The heterogeneity in findings observed across different models reflects the intrinsic heterogeneity of human TBI and suggests that both the magnitude and specific biomechanical characteristics of the blast wave are important factors in influencing the pathophysiological pathways activated after injury [40]. For example, in microfluidic flow chamber models, calcium influx is inhibited by Gd^3+^, but not VGCC inhibitors, whereas in microcavitation models, the opposite is observed and VGCC inhibition, but not Gd^3+^, reduces calcium influx [34,40]. This suggests that different blast parameters, including blast intensity, can activate divergent physiological pathways. In some studies, low-magnitude blasts caused no significant disruption in calcium homeostasis, and cells returned to baseline activity within an hour [36,45]. In other studies, moderate blast injury, in contrast to higher-magnitude blasts, was associated with increased expression of Piezo 2, a mechanosensitive calcium channel regulator [44]. High-magnitude injury in vitro consistently resulted in elevated intracellular calcium levels and disrupted calcium spiking activity [34,37,41,65]. In combination, these findings suggest that threshold and dose dependencies influence altered calcium signaling relative to blast intensity and that shear strain is important in both in vivo and in vitro models, underscoring the complexity of blast-induced neurotrauma and potentially accounting for some of the heterogeneity observed in animal models [69].

### 3.2. Endoplasmic Reticulum Stress and Astrogliosis Related to bTBI

A discussion of the molecular neuropathology of TBI must include endoplasmic reticulum (ER) stress and proteostasis, processes implicated in chronic neurodegeneration and inflammation. These mechanisms include the unfolded protein response (UPR), ER-associated degradation (ERAD), autophagy, and cellular oxidative stress [72,73,74]. The ER, a membrane-bound organelle essential for protein and lipid synthesis, plays a critical role in maintaining cellular homeostasis and calcium signaling [75,76]. ER calcium stores, regulated by inositol-1,4,5-trisphosphate (IP3)-sensitive receptors (IP3Rs), ryanodine receptors (RyRs), and the sarcoplasmic/endoplasmic reticulum calcium ATPase (SERCA) pump, are fundamental to calcium signaling and homeostasis [77].

IP3Rs are activated via binding IP3, which is produced by a G-protein-coupled signaling cascade to trigger calcium release from the ER in response to extracellular signals [78]. In non-muscle cells, ryanodine receptors (RyRs) are usually activated through calcium-induced calcium release (CICR), where localized calcium elevations in proximity to RyRs stimulate further calcium release from ER stores, creating a positive feedback loop [79]. These mechanisms are critical for coordinating intracellular calcium signaling. The ER also facilitates post-translational modifications essential for correct protein folding and function. Because misfolded proteins can accumulate and disturb normal cellular function, the UPR plays a critical role in protein clearance and cell function. That is, the ER promotes protein refolding and degradation of misfolded proteins to restore homeostasis. However, if ER stress is prolonged, it shifts towards apoptotic signaling [80]. Accumulation of misfolded proteins is a pathological hallmark of neurodegenerative disease, including trauma-related neurodegeneration [81].

In astrocytes, prolonged UPR activation is associated with reactive astrocytosis, a non-specific pathological response common to TBI and other neurological disorders that is characterized by astrocyte hypertrophy and upregulation of glial-specific proteins such as glial fibrillary acidic protein (GFAP) [82]. Astrocytes are integral to the maintenance of the blood–brain barrier, synaptic function, and neurotransmitter recycling [83,84,85,86]. Although astrocytic activation is an important initial response to injury that supports neuronal survival, chronic gliosis can contribute to neuroinflammation and neurodegeneration [87].

Simulations of bTBI in primary astrocyte cultures result in increased intracellular calcium due to CICR from intracellular stores [40]. When extracellular calcium is removed, intracellular calcium chelated, or ER calcium stores depleted, neurons and astrocytes fail to demonstrate post-injury calcium increases [34,40,65]. This indicates that mechanisms such as mechanoporation, VGCCs, and mechanosensitive calcium channels can synergize with ER calcium stores to drive rapid intracellular changes in calcium after blast exposure. In mixed cultures, repeat blast injury induces significant changes in neurons, but not in the overall mixed cultures, suggesting that glial cells may buffer calcium to mitigate some of the detrimental effects on neurons [40]. In astrocytes, this calcium surge may function as an early injury response signal, triggering subsequent signaling cascades that lead to astrogliosis [40]. Further, astrocytic volume post-injury is modulated by mechanosensitive pathways involved in calcium uptake, with calcium-permeable channels including TRPV4 and L-type VGCCs showing upregulation in reactive astrocytes [88,89,90]. Other in vitro blast models have demonstrated that increased astrocytic calcium post-injury correlates with upregulated GFAP expression [34,42]. Similarly, transcriptomic profiling of rat hippocampus performed 24 h post-blast exposure demonstrated increased expression of GFAP and vimentin with decreased expression of calcium-signaling-associated genes such as CaMKIIb, CaMKIInI, and calreticulin [39]. Studies using brain homogenates from Yucatan miniature and Yorkshire swine have demonstrated increased GFAP and vimentin deimination (citrullination) after blast exposure [33]. This calcium-dependent post-translational modification, where arginine is converted to citrulline, may render proteins immunogenic and potentially contribute to post-traumatic autoimmune-mediated neurological dysfunction, analogous to other autoimmune conditions such as rheumatoid arthritis [91]. Notably, non-blast TBI has been associated with brain-specific antibodies against GFAP [92], suggesting additional potential links between deiminated proteins, astrogliosis, and autoimmunity [33]. Although human data are limited, military blast exposures are associated with prominent and distinctive patterns of interface astrocyte activation that differ morphologically from other forms of TBI, suggesting potentially unique astrocytic responses after blast exposure [93,94].

The interplay between ER, plasma membrane calcium signaling, and reactive astrocytosis further underscores the complexity of cellular pathways activated after LLB exposure. Astrocytes function as dynamic interconnected networks with intricate connections among cells [95]. After blast injury, elevated calcium in astrocytes propagates as waves to neighboring astrocytes, even those not directly affected by the insult [42]. This wave propagation can be blocked by P2 antagonists. Targeting CICR and associated ER-related mechanisms could potentially limit astrocytic inflammatory responses, mitigate neurological dysfunction, and promote recovery following LLB exposure.

### 3.3. Cytoskeletal Breakdown and Mitochondrial Dysfunction Post bTBI

Rapid spikes in intracellular calcium after blast exposure and mechanical shear can overwhelm the cells’ buffering capacity [96]. To restore calcium homeostasis, calcium-binding proteins act as immediate buffers, and then ion pumps such as plasma membrane calcium ATPase (PMCA) and SERCA return excess calcium to the extracellular space or intracellular stores [97]. Failure to appropriately regulate these changes in calcium levels can lead to enzymatic activation, mitochondrial dysfunction, and apoptosis [98,99].

The cytoskeleton, composed of microtubules, neurofilaments, and actin filaments, is especially vulnerable to calcium dysregulation following blast. Tau, a microtubule-associated protein predominantly found in neurons, stabilizes microtubules and supports axonal transport under normal conditions [100]. However, pathological changes in tau such as hyperphosphorylation and aggregation as neurofibrillary tangles results in destabilization of microtubules and the cytoskeleton [100,101]. Repeat TBI is linked to several tauopathies, including Alzheimer’s disease and chronic traumatic encephalopathy (CTE) [102,103,104,105,106,107,108].

Calpain, a calcium-activated protease, plays a central role in cytoskeletal degradation after TBI [32]. In chick forebrain neurons, calpain is activated by calcium influx following axonal mechanoporation, which can be blocked by the calpain inhibitor ALLN [65]. Calpain activity often co-localizes with mitochondria and is associated with the formation of axonal swellings, a neuropathological hallmark of diffuse axonal injury [65]. Liquid chromatography–mass spectrometry proteomic profiling of brain tissue homogenates from blast-injured mice has demonstrated an upregulation of proteins linked to axonal injury and tau pathology after a single open-field blast exposure [35]. In particular, cytoskeletal structural proteins such as Ablim1, neurofilament medium chain (NEFM), and plectin were initially downregulated at 3 and 24 h post-injury, but later showed partial recovery [35]. Additionally, phosphorylated tau (p-tau) levels and p-tau/tau ratios were significantly increased within the first 24 h post-injury, but gradually returned to sham-treated levels by 15 weeks.

In contrast, when brain tissue from a rat model of bTBI was evaluated for tau pathology using antibodies against AT8 and pan-tau, there was no significant change in tau expression for up to 2 weeks post-blast, indicating that tau pathology may be influenced by additional factors such as the blast injury model used, severity of BOP exposure, and the animal species evaluated [39,109]. Human data are limited, but tau pathology does not appear to be a prominent feature in the brains of deceased service members with prior blast exposures [110].

Mitochondrial dysfunction associated with disrupted calcium homeostasis after TBI is thought to lead to metabolic supply-demand mismatch and contributes to secondary brain injury mechanisms [32,111]. Under normal physiological conditions, calcium stimulates mitochondrial enzymes involved in the Krebs cycle and electron transport chain (ETC) to enhance ATP production [112,113]. However, excessive calcium can overload the mitochondria, resulting in mitochondrial swelling, disruption of the ETC, and decreased ATP synthesis [114]. Prolonged overload triggers apoptosis through the release of pro-apoptotic factors such as cytochrome c into the cytoplasm [115,116]. Additionally, increased mitochondrial calcium promotes the production of reactive oxygen species (ROS) [117].

Blast-induced calcium dysregulation can directly impair mitochondrial function. In chick forebrain neurons, the accumulation of calcium in injured axons after blast exposure demonstrates peaks that co-localize with mitochondria, highlighting the importance of mitochondria in calcium buffering and homeostasis [65]. Rats exposed to low- and moderate-intensity blasts show upregulation of antioxidant enzymes, including super oxide dismutase (SOD), associated with increased Piezo 2 expression, suggesting a mitochondrial response that is linked to mechanotransduction-induced changes in calcium homeostasis [44]. However, higher-pressure blasts downregulate expression of antioxidant enzymes, suggesting oxidative damage at higher intensities can overwhelm cellular repair mechanisms [44].

Temporal studies highlight a progressive failure of cellular responses post-blast. In a rat model of bTBI, reduced glutathione levels and increased lipid peroxidation were observed at 2 and 24 h post-injury, reflecting oxidative stress [39]. Interestingly, even low-magnitude blasts that do not significantly alter calcium homeostasis can induce ROS production in cultured rat neurons, suggesting that calcium-independent pathways are also important for ROS production [45].

Mice exposed to a single open-field blast demonstrated increased expression of the mitochondrial ADP-ATP exchanger, presumably in response to reduced ATP levels [35]. However, variations in metabolic responses were observed across different blast injury models. For example, ATP levels remained stable, but purine metabolism was disrupted in rats in a shock-tube blast model, potentially reflecting species-specific vulnerabilities across different signaling pathways [39]. In contrast, open-field full-body blast exposures in mice resulted in markedly compromised ATP metabolism, again underscoring striking variation based on the blast injury model, the intensity of BOP exposure, and the animal species evaluated.

The consistent observation of altered ROS production across different in vivo and in vitro models of bTBI highlights a central role for ROS in blast-induced neurotrauma [39,44,45]. Without intervention, a vicious cycle emerges, whereby plasma membrane disruption leads to a calcium influx and the production of ROS, which drives lipid peroxidation, resulting in further membrane disruption. Targeting this vicious cycle is a potential way to mitigate the cellular energy failure that follows TBI.

### 3.4. Nuclear Calcium Signaling After bTBI

Nuclear calcium channels play an important role in regulating cellular processes such as gene transcription, proliferation, and apoptosis [118,119]. In neurons, nuclear calcium channels facilitate the conversion of synaptic inputs and neuronal activity into specific transcriptional programs [118]. In non-blast TBI, when other buffering systems fail, cytosolic calcium may be sequestered into the nucleus, compromising nuclear calcium homeostasis and leading to the activation of nucleases that degrade DNA [32].

Primary regulators of communication between cytosolic calcium and nuclear transcription are CaMKII and CaMKIV, which are activated by nuclear calcium–CaM complexes leading to phosphorylation and activation of transcription factors such as CREB and CBP, which in turn influence gene expression [120,121]. Blast injury models have yielded conflicting patterns in terms of the expression of these proteins. A rat model showed a decrease in CaMKIIb and CaMK2nb following a blast, whereas a mouse model showed increased phosphorylation of CaMKIIb and CaMKIV at multiple time points post-injury (3, 24 h, and 15 weeks) [35,39]. Recent advances in spatial genomic technologies offer an opportunity for improved clarification of the roles of CaMKIIb and CaMKIV, chromatin remodeling, and the expression of other nuclear proteins as potential targets for therapeutic intervention after blast exposure.

Blast injury has been linked to increased expression of nuclear genes involved in mitochondrial respiration, including COX7b, Ndufa11, and Uqcrh, which contrasts with the downregulated gene expression previously reported in controlled cortical impact models, suggesting that blast exposure may exert different effects on mitochondrial gene function compared to other forms of TBI [39,122].

### 3.5. Lysosomal Pathways Related to bTBI and Neurodegeneration

Lysosomes, essential for cellular homeostasis, are involved in the autophagy-dependent degradation pathway, responsible for clearing cellular debris through fusion of autophagosomes with lysosomes [123,124,125]. Perturbations of this pathway are implicated in chronic TBI pathology [125,126]. In chronic neurodegenerative disorders such as Alzheimer’s disease, calcium dysregulation can disrupt autophagic clearance [127]. Transcriptomic analysis of hippocampal gene expression in a rat model of bTBI demonstrated underexpression of lysosomal genes such as lysosomal membrane glycoprotein (Lamp1) and lysosomal-associated protein transmembrane 4a (Laptm4a), suggesting potential lysosomal dysfunction [39]. However, the precise connection between lysosomes and calcium homeostasis in bTBI remains unclear, and further research is necessary to elucidate the nature of this relationship.

## 4. Mechanistic Insights and Therapeutic Targets

Loss of calcium homeostasis and dysregulated calcium signaling are hallmark features of bTBI pathophysiology, particularly when associated with cellular shear. This review emphasizes the heterogeneity of mechanisms that can potentially lead to calcium dysregulation after blast exposure, underscoring that cellular pathology is mediated through a complex interplay of multiple different mechanisms rather than a single causative event. These mechanisms involve multiple distinct cellular compartments and include mechanoporation, calcium channel dysfunction (both VGCCs and mechanosensitive channels), intracellular release from ER stores, mitochondrial dysregulation, and lysosomal pathway stress. The collective contributions of these separate processes drive downstream pathophysiological processes including astrocytosis, neuroinflammation, cytoskeletal instability, degeneration, and protein misfolding.

The heterogeneity in potential cellular responses to injury poses a major challenge for developing effective therapies. In the studies reviewed, striking variability was observed even among cells of the same type, as demonstrated by concurrent increases and decreases in neuronal calcium across hippocampal networks after bTBI [36]. Additionally, distinct patterns of calcium dysregulation were evident across different cell types. For example, astrocytes predominantly exhibited calcium-mediated responses through purinergic signaling, the ER, and astrocytosis, whereas neurons relied heavily on VGCC-mediated and mechanosensitive channel calcium influx, leading to cytoskeletal degradation and mitochondrial dysfunction, although convergence of these different mechanisms was also common. These differences underscore the distinct roles of different cell types in central nervous system pathology and emphasize the need for developing target and cell-specific therapies. Despite these differences, there are also some mechanistic points of convergence. For example, mechanoporation emerges as a common pathway across cell types, making it a promising therapeutic target, and P188, a membrane-sealing agent, has shown promise in restoring calcium homeostasis post-injury. Other key compounds that have been used to target calcium signaling after blast exposure are summarized in Table 2, further underscoring the importance of considering cellular heterogeneity in experimental design.

The effects of variation become even more pronounced when evaluating animal models of bTBI. Significant differences exist in the design and construction of shock tubes used to generate experimental blasts, the selection and administration of anesthetics, and the anatomic and physiologic fidelity of the animal model (e.g., lissencephaly in rodents versus gyrencephaly in ferrets and larger animals) [36]. Additional factors such as the orientation of the animal with respect to the blast, lack of consensus on appropriate blast intensity and frequency, and variability in recovery conditions post-blast further complicate reliable comparisons across studies. Both in vitro and in vivo bTBI models have demonstrated that repetitive injuries can lead to cumulative detrimental outcomes compared to a single blast, underscoring the necessity of distinguishing between single and repeat exposures [36,45]. In combination, these variations in experimental design have significantly hampered translational relevance for human bTBI and have contributed to inconsistencies in behavioral and neuropathological outcomes across laboratories [69]. Standardizing these parameters is urgently needed to improve model reproducibility and facilitate the identification and validation of potential bTBI biomarkers and therapeutic targets before translation into clinical contexts, where additional biological variables such as patient sex, brain developmental stage, and medical comorbidities further increase variability.

Much remains unknown about the long-term sequelae of repeat LLB exposure, particularly the risk of later Alzheimer’s disease and chronic traumatic encephalopathy (CTE). Decades of clinical research have established robust epidemiological links between non-blast forms of TBI and Alzheimer’s disease. Systematic reviews suggest that the lifetime risk of dementia increases by 63–96% after a moderate to severe TBI [128]. For veterans with mild TBI, the risk doubles, whereas moderate to severe TBI has been associated with a four-fold increase in dementia risk [129,130,131,132]. A previous Cochrane review found insufficient evidence to support the use of calcium channel blockers in acute TBI, although this review oversimplified the heterogeneity of TBI and did not specifically account for blast-related exposures [133]. The lack of standardization in experimental models and variability in injury mechanisms has further limited clear conclusions regarding their pharmacologic effects. Revisiting calcium channel blockers and buffers with more targeted injury-specific approaches relevant to bTBI may yield more promising results.

One potential therapeutic strategy moving forward could be to adopt a precision medicine-based approach modeled after neuro-oncology, thereby allowing for personalized therapies that capture the dynamic and multifaceted nature of bTBI. For instance, therapies that target the ER and glial inflammation might be more effective in injuries with astrocytic calcium dysregulation as the dominant pathology. In contrast, targeting mitochondrial stabilization to limit metabolic deficits and oxidative stress might be more effective in cases where mitochondrial dysfunction is the primary phenotype. For axonal injuries, membrane-stabilizing agents such as P188 might be more effective. Such an approach would require improved diagnostic, predictive, and prognostic TBI biomarkers that better capture the nuances of clinical endophenotypes to enable these distinctions and to guide improved diagnostics and therapy [134].

## 5. Future Directions

Recent advances in spatial genomics, single-cell RNA sequencing, and advanced neuroimaging techniques provide powerful tools to characterize bTBI at the metabolic, cellular, and molecular scales. These technologies offer enhanced resolution at the tissue level, enabling a more detailed understanding of key pathological processes such as calcium dysregulation, mitochondrial dysfunction, and cytoskeletal breakdown within neuronal networks after bTBI.

However, to facilitate the development of new therapeutic strategies, it will also be important to address the challenges posed by a lack of standardized operational definitions, as well as the variability, in the fidelity and evaluation of blast injury models. Standardization of these efforts coupled with the incorporation of biological variables such as age and sex will significantly improve the translational relevance of preclinical findings. A deeper understanding of the acute and long-term effects of bTBI on dysregulated calcium signaling, as well as the subsequent risk for chronic neurodegenerative diseases such as Alzheimer’s disease, is critical. With new information, it will be possible to identify the roles of specific calcium channels in leading to downstream sequelae of bTBI. And with the identification of calcium channels as cellular targets, new treatments can be developed. In the short term, identifying biomarkers that reflect blast-induced calcium dysregulation could improve early diagnosis of subtle neuropsychological dysfunction and allow for precision-based interventions. Integrating these biomarker data into preclinical drug screening studies could also increase the likelihood of translating preclinical discoveries into effective therapeutics [134].

## 6. Conclusions

Blast TBI is a complex condition associated with loss of calcium homeostasis, mechanoporation, mitochondrial dysfunction, cytoskeletal instability, and disrupted nuclear calcium signaling. The potential long-term effects of blast exposure are of increasing concern for service members [135,136]. Recurrent low-level blast exposures are associated with distinct neuropathological and clinical features, underscoring the need for focused research in this area. However, variations in experimental design and classification remain significant barriers to progress. Efforts at standardization, supported by emerging technologies like spatial genomics and advanced neuroimaging, hold promise for improving biomarker identification and advancing precision-based treatments.

## Figures and Tables

**Table 1:** Changes in Ca^2+^ signaling in vivo/in vitro.

Type	Species	Brain Region	Cell Type	[Ca^2+^]	Major Finding	Reference
VV	Pig	Frontal lobe	Mixed	N/A	bTBI promotes aberrant protein deimination by Ca^2+^-dependent enzymes	[33]
VT	Mouse	Cerebellum	Astrocyte	**↑**	Poloxamer 188 and ω-conotoxin rescue disrupted Ca^2+^ homeostasis	[34]
VV	Mouse	Entorhinal cortx, white matter, cerebral peduncle	Astrocyte	N/A	Upregulation of Ca^2+^ signaling pathway-associated proteins	[35]
VV	Mouse	Hippocampus CA1	Neuron	**↓**	bTBI induces a reduction in slow and fast Ca^2+^ dynamics	[36]
VT	Mouse	Cerebellum	Astrocyte	**↑**	Poloxamer 188 partially rescues calcium homeostasis and reduces ROS production	[37]
VT	Chick	Embryonic day 8 forebrain	Neuron	**↑**	Mechanoporation leads to Ca^2+^ increase and calpain activation	[38]
VV	Rat	Hippocampus, frontal lobe	Mixed	N/A	Downregulation of Ca^2+^ signaling genes at 24 h post-blast	[39]
VT	Rat	Site not specified	Astrocyte	**↑**	Increased intracellular Ca^2+^ is blocked by mechanosensitive but not VGCC blockers	[40]
VT	Human	Site not specified	Dissociated CNS cells	**↑**	Shear forces, not blast injury alone, disrupt Ca^2+^ signaling	[41] *
VT	Human, Rat	Site not specified	Dissociated CNS cells	**↑**	Ca^2+^ waves propagate by purinergic signaling in astrocytes	[42] *
VT	Human	Site not specified	Dissociated CNS cells	**↑**	Shear forces are sufficient to disrupt Ca^2+^ signaling with minimal pressure changes	[43] *
VV	Rat	Hippocampus	Mixed	N/A	Increased Piezo 2 expression after blast exposure	[44]
VT	Rat	Cerebrum	Neuron Mixed	↔	membrane permeability increased by blast and Na+ influx after blast, no significant change in Ca^2+^	[45]

VV: in vivo; VT: in vitro; Ca^2+^: cytoplasmic free Ca^2+^; N/A: not applicable. * indicates papers originating from the same group. up arrow = increased response, down arrow = decreased response, horizontal arrow = no significant change.

**Table 2:** Pharmacological compounds tested in vitro.

Experimental Design	Compound	Mechanistic Background	Reference
Microcavitation in mouse astrocytes	Poloxamer 188, ω-conotoxin	Ca^2+^ spiking partially rescued by both reagents individually, no additive effects in combination	[34]
Microcavitation in mouse astrocytes	Poloxamer 188	Ca^2+^ spiking partially rescued	[37]
Mechanical membrane injury in chick embryonic neurons	Poloxamer 188	Intracellular Ca^2+^ spiking blocked, probably by sealing membrane pores	[65]
Shear stress in rat astrocytes	Gd^3+^	Ca^2+^ influx in astrocyes inhibited by mechanosensitive channel blocker	[40]
Dissociated human cultures exposed to simulated blast	PPADS	Significantly reduced Ca^2+^ response in astrocytes without affecting neuronal response	[71]

This table shows the effects of pharmacological compounds that were tested and their effects on injured cells, representing several in vitro models.

## Data Availability

This is a review study of the published literature, and no new data were created. All data supporting the reported findings are available in the referenced publications.
